# Mesenchymal stem cells contribute to the chemoresistance of hepatocellular carcinoma cells in inflammatory environment by inducing autophagy

**DOI:** 10.1186/2045-3701-4-22

**Published:** 2014-04-28

**Authors:** Zhipeng Han, Yingying Jing, Yong Xia, Shanshan Zhang, Jing Hou, Yan Meng, Fenghai Yu, Xiaoqing Liu, Mengchao Wu, Ping Zhang, Lixin Wei

**Affiliations:** 1 Tumor Immunology and Gene Therapy Center, Eastern Hepatobiliary Surgery Hospital, the Second Military Medicial University, 225 Changhai Road, Shanghai 200438, China; 2 Department of Comprehensive Treatment, Eastern Hepatobiliary Surgery Hospital, the Second Military Medicial University, Shanghai, China; 3 Department of Radiation Oncology, Eastern Hepatobiliary Surgery Hospital, the Second Military Medical University, Shanghai, China; 4 Department of Gastroenterology, Eastern Hepatobiliary Surgery Hospital, the Second Military Medical University, Shanghai, China; 5 Department of Ophthalmology, Shanghai Tenth People’s Hospital and Tongji Eye Institute, Tongji University School of Medicine, Shanghai, China; 6 Department of Surgery, College of Human Medicine, Michigan State University, East Lansing, MI, USA

**Keywords:** Mesenchymal stem cells, Inflammation, Autophagy, Hepatocellular carcinoma

## Abstract

**Background:**

Mesenchymal stem cells (MSCs) have been reported to play an important role in tumor growth. Inflammation is an important feature of hepatocellular carcinoma (HCC). Certain inflammatory cytokines produced in tumor microenvironment modulate functional activities of MSCs. At the present time, however, the role of MSCs in the development of HCC cell resistance to chemotherapy in the inflammatory microenvironment during tumor growth has not yet been identified.

**Methods:**

MTT and PI/Annexin V-FITC assay were employed to examine the proliferation and apoptosis of HCC cell lines. The expression of TGF-β are detected by Realtime PCR and Western blot. GFP tagged LC3 expression vector and electron microscopy are utilized to demonstrate the occurrence of autophagy.

**Results:**

We observed that MSCs pretreated with the combination of IFN-γ and TNF-α induced resistance to chemotherapy in HCC cell lines in both the *in vitro* and *in vivo* circumstances. Following exposure to conditioned medium of MSCs that were pre-treated with IFN-γ plus TNF-α, HCC cell line cells underwent autophagy which serves as a protective mechanism for HCC cells to resist the cell toxicity of chemotherapeutic agents. Treatment of HCC cell line cells with autophagy inhibitor effectively reversed the MSCs-induced resistance to chemotherapy in these cells. Stimulation with the combination of IFN-γ and TNF-α provoked expression of TGF-β by MSCs. MSCs-induced chemoresistance in HCC cell lines was correlated with the up-regulation of TGF-β expression by MSCs. Knockdown of TGF-β expression by MSCs with siRNA attenuated MSCs-induced chemoresistance in HCC cells.

**Conclusions:**

These results suggest that increase in TGF-β expression by MSCs in the inflammatory microenvironment of HCC promotes the development of chemoresistance in HCC cells.

## Background

Chemotherapy remains a major treatment alternatively to surgery for a large number of patients at the advanced stage of hepatocellular carcinoma (HCC). However, development of chemoresistance in HCC cells becomes a prominent obstacle for effective treatment of HCC with chemotherapy. In addition to the chemoresistance originated from HCC cells themselves, accumulated evidences suggest that tumor microenvironment may also play an important role in inducing and/or promoting the development of drug resistance. Studies have shown that existence of certain specific properties of stromal cells is an indicator for poor prognosis [1]. Stromal cells may modulate the reaction of tumor cells to chemotherapy in various ways [2-5].

Mesenchymal stem cells (MSCs) have caught a wide attention in recent years because of their therapeutic potential to treat human diseases and their physiological roles *in vivo*[6,7]. MSCs are originally found in the bone marrow, but they have also been isolated from other sites in the body such as adipose tissue and uterus [8]. MSCs have the ability to differentiate into cells of multiple lineages including chondrocytes, osteocytes, adipocytes, myocytes, and astrocytes [9,10]. MSCs is positive for CD29, CD90 and CD105 and is negative for hematopoietic cell markers CD34 and CD45 [11]. MSCs express the major histocompatibility complex (MHC) class I but do not express MHC class II, B7-1, B7-2, CD40, or CD40L molecules. They can be expanded more than 10^4^-fold in culture without losing multilineage differentiation potential [12]. Therefore, MSCs are not only considered as an acceptable source of stem cells in hematopoietic recovery, but also an available cell type for tissue regeneration [9].

Interestingly, MSCs have the ability to migrate to tumor sites in different types of tumor models [13-15]. Inflammation is a common feature of the tumor microenvironment [16], which consists of cytokines, chemokines, and infiltrated leukocytes. MSCs may secrete several different types of mediators including interleukin-10 (IL-10) [17], transforming growth factor-β (TGF-β) [17,18], hepatocyte growth factor (HGF) [18], and vascular endothelial growth factor (VEGF) [19] when exposed to inflammatory cytokines, which has been reported to play an important role in tumor development.

In previous studies, we have demonstrated that MSCs in inflammatory environment may promote tumor growth via immunosuppression and angiogenesis. However, the role of MSCs in the development of chemoresistance in HCC cells in the inflammatory microenvironment during tumor growth remains unclear. The purpose of this study is to investigate the contribution of MSCs to development of chemoresistance in HCC cells and its potentially underlying mechanism.

## Methods

### Reagents

Recombinant human IFN-γ (Cat. 300-02B) and TNF-α (Cat. 300-01A) were obtained from Peprotech (La Jolla, CA). Stock solutions of chemotherapeutic agents cisplatin (Qilu Pharmaceutical Co., Ltd., Jinan, Shandong, China, Cat.H20073653) and epirubicin (Pfizer Pharmaceuticals Limited, Wuxi, Jiangsu, China, Cat.H20093251) were prepared at the concentration of 1 mM, respectively. The stock solution of each agent was then added directly into cell culture media to achieve final concentrations as indicated in each figure legend in the “Results” section. Chloroquine (CQ, Cat.C6628) and 3-methyladenine (3-MA, Cat.M9281) were purchased from Sigma-Aldrich (St. Louis, MO).

### Culture of cell line cells

Human MSCs (a gift from Institute of Health Sciences and Shanghai Institute of Immunology, Chinese Academy of Sciences, Shanghai, China) were cultured in Dulbecco’s modified Eagle’s medium (DMEM) nutrient mix F12 with fetal bovine serum (FBS, 10%; Invitrogen, Cat. 10099-054). HCC cell line cells including SMMC-7721 cells and Hep-G2 cells were cultured in DMEM containing 10% FBS. All cells were cultured at 37°C in a humidified atmosphere containing 5% CO_2_.

### Generation of conditioned medium

MSCs were stimulated with IFN-γ (20 ng/ml), TNF-α (20 ng/ml) or both for 12 h. The culture medium of MSCs was removed. Stimulated MSCs were cultured in fresh DMEM nutrient mix F12 for 24 h. The conditioned medium in the MSC culture was then collected and filtered through a 0.22 μm filter.

### Real-time RT-PCR

MSCs were cultured with IFN-γ (20 ng/ml), TNF-α (20 ng/ml), or both for 12 h. The cells were collected. Total mRNA was extracted from cells using the Trizol Reagent (Invitrogen, Carlsbad, CA, Cat.15596-026) and protocol provided by the manufacturer. Expression of TGF-β mRNA was determined by real-time RT-PCR using the SYBR Green Master Mix Kit (Applied Biosystems, Foster City, CA, Cat.4306736) and protocol provided by the manufacturer. The amount of endogenous β-actin mRNA was used for normalization of TGF-β mRNA expression in each sample. Primers for TGF-β mRNA determination were: 5′-GCCGAGCCCTGGACACCAAC-3′ (forward) and 5′-GCGCCCGGGTTATGCTGGTT-3′ (reverse). Thermocycler conditions included an initial hold at 50°C for 2 minutes and then 95°C for 10 minutes, which was followed by a two-step PCR program of 95°C for 15 seconds and 60°C for 60 seconds repeated for 40 cycles on a Mx4000 system (Stratagene). Alteration of TGF-β mRNA expression is presented as the fold change relative to an untreated control.

### MTT colorimetric assay

To measure the effects of MSCs on chemosensitivity of human hepatoma cells, HCC cells were seeded in 96-well plates at a density of 1 × 10^4^ cells/well and cultured in the medium containing chemotherapeutic agents with or without MSCs conditioned medium for 8 h. The cell viability in each well was examined by a MTT (5 mg/ml, Sigma-Aldrich, Cat.M2003) colorimetric assay. The optical density (OD) value at 490 nm of each sample was measured using a plate reader. All determinations were carried out in sextuplicate.

### Cell apoptosis assay

HCC cells (2 × 10^5^cells/well) were cultured in 6-well plates to 70–80% confluence. The cells were then treated with chemotherapeutic agents for 8 h in the absence and presence of MSC conditioned medium. In a subset of experiments, 3-MA (5 mM) was used to block autophagy. PI/Annexin V-FITC assay was used to measure apoptotic cells by flow cytometry according to the manufacturer’s instruction (Keygen Biotech. Co., Ltd, Nanjing, Jiangsu, China, Cat.KGA108). Briefly, cells collected by trypsinization were washed trice with ice cold phosphate-buffered saline (PBS). Cells were then incubated in 300 μL of 1× binding buffer containing 5 μL Annexin V and 5 μL PI for 30 min at room temperature in the dark. Apoptosis of cells was measured on a BD FACSAria flow cytometer (Becton Dickinson, Lincoln Park, NJ). At least 30,000 gated events were acquired from each sample. Results are expressed as the percentage of apoptotic cells (PI and Annexin V positive) in the gated cell population.

### Transient transfection and identification of autophagy

GFP tagged LC3 expression vector has recently been utilized to demonstrate the occurrence of autophagy. SMMC-7721 and Hep3B cells were seeded (1 × 10^4^ cells/well) in 96-well plates overnight. GFP-LC3 expressing plasmids were transiently transfected into cells using the Fugene HD transfection reagent (Roche, NSW, Australia, Cat.04709705001) according to the manufacturer’s instruction. After being cultured for 24 h to ensure expression of GFP-LC3, the cells were treated with MSC conditioned medium and cisplatin for 8 h. In a subset of experiments, 3-MA was also added to the cell culture. At the end of each experiment, autophagy was detected by counting the number of cells with GFP-LC3-positive dots under fluorescence microscope (Olympus IX71). A minimum of 200 cells were counted in each sample. The experiment was conducted in triplicate.

### Electron microscopy

HCC cells were sequentially fixed with 2.5% glutaraldehyde acid in 0.1 M PBS buffer (pH 7.4) for 2 h, incubated in 1% osmium tetroxide in 0.1 M PBS buffer (pH 7.4) for 2–3 h, dehydrated in solutions of ethanol and acetone, embedded in Araldite, and finally solidified. Sections (50-60 nm) were cut on a LKB-I ultramicrotome and picked up on copper grids. After being post-stained with uranyl acetate and lead citrate, sample sections were observed with a Philips CM-120 TEM (Philips).

### Animal model

All procedures involving animals were performed in accordance with the institutional animal welfare guidelines of Second Military Medical University. Subcutaneous implantation of HCC cells (alone or mixed with MSCs) was performed in armpit areas of nude mice. Mice were examined three times per week. Tumor growth was evaluated by measuring the length and width of the tumor mass. Animals were sacrificed and tumors were removed at the end of the experiment. Tumor masses were weighed and analyzed by histology.

### Enzyme linked immunosorbent assay

ELISA assays were performed with a commercial TGF-β1 ELISA kit (R&D Systems, Minneapolis, MN). Conditioned medium was collected from wells, aliquoted, and stored frozen. Assays were performed in duplicate, and readings were compared with standard curves obtained with standard protein provided with the kit. Means and standard deviations of concentrations in triplicate samples were compared by *t*-test.

### Western blot analysis

Extraction of total soluble proteins from cultivated cells and western-blot analysis were performed as previously described [20]. Primary antibodies used in western blot analysis were specific for either TGF-β (Abcam, Hongkong, China, Cat.ab66043) or β-actin (Invitrogen, Cat. AM4302). Horseradish peroxidase conjugated goat anti-rabbit IgG was used as the secondary antibody (R&D systerms, Cat. BAF008).

### Short interfering RNA (siRNA) synthesis and transient transfection

Three siRNA sequences of TGF-β1 were designed using Oligoengine software and verified by nucleotide BLAST searches. Three candidate sequences and a control sequence with no significant homology were listed in Table 1. Cells (1-3 × 10^6^) growing to 50%-60% confluence in 10 cm petri dishes were transfected with siRNA sequences or their corresponding mock sequences using a Lipofectamine 2000 kit (Invitrogen, Cat.11668-019) with the procedure provided by the manufacturer. Cells were observed under a fluorescence microscope and harvested 48 h after transfection.

**Table 1:** Sequence of the oligonucleotides for siRNA construct-making assays

**Assays**	**Gene**	**Sequence (5′ → 3′)**	
**TGFβ1 siRNA**	Sequence 1	Sense	CACUGCAAGUGGACAUCAATT
		Antisense	UUGAUGUCCACUUGCAGUGTT
	Sequence 2	Sense	GCAAGACUAUCGACAUGGATT
		Antisense	UCCAUGUCGAUAGUCUUGCTT
	Sequence 3	Sense	GCAUAUAUAUGUUCUUCAATT
		Antisense	UUGAAGAACAUAUAUAUGCTT
	Control	Sense	UUCUCCGAACGUGUCACGUTT
		Antisense	ACGUGACACGUUCGGAGAATT

### Statistical analysis

All data, expressed as mean ± SEM, were from at least three separate experiments. Data sets were analyzed by analysis of variance (ANOVA) with a posteriori contrast by least significant difference (for comparisons among multiple groups) or by Student *t*-test (for comparison between two groups) using the Microsoft Excel Analysis Tool Pak (Microsoft, Redmond, WA). P < 0.05 was considered to be statistically significant.

## Results

### Inflammatory cytokine-stimulated MSCs enhanced chemoresistance of hepatocellular carcinoma cells

**Figure 1:**
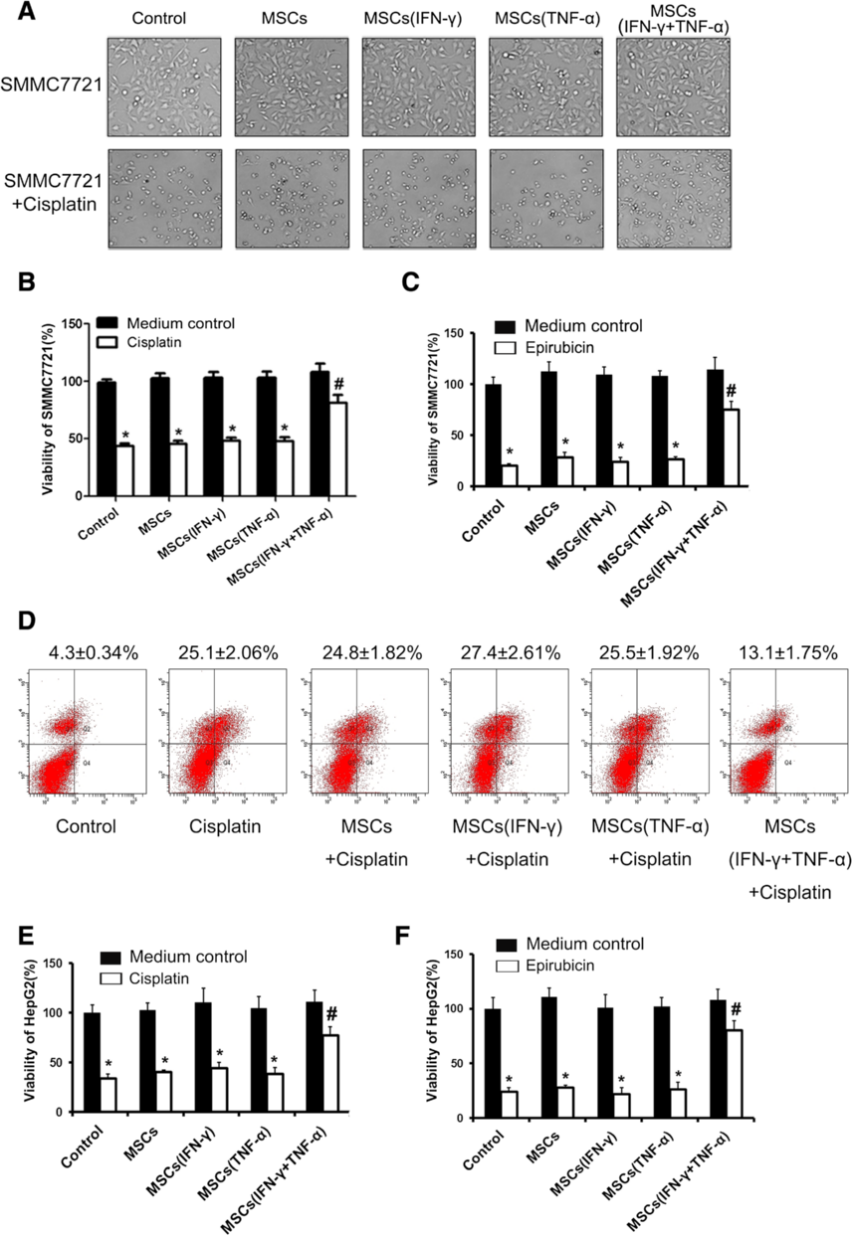
**Conditioned medium from the culture of inflammatory cytokine-stimulated MSCs enhanced chemoresistance of HCC cells *in vitro. *(A)** SMMC-7721 cells were treated with cisplatin (20 μM) with conditioned medium collected from MSCs which were pretreated with inflammatory cytokines or not. The morphology of the cells was captured by microscope. MTT was employed to examine the proliferation of SMMC-7721 cells, which were treated with cisplatin (20 μM) **(B)** or epirubicin (0.55 μg/ml) **(C)** with conditioned medium collected from MSCs which were pretreated with inflammatory cytokines or not. **(D)** SMMC-7721 cells were cultured in a 6-well plate with an existence of cisplatin (20 μM) and the conditioned medium collected from MSCs which were pretreated with inflammatory cytokines or not were added in SMMC-7721 culture medium for 24 hours. PI/Annexin V-FITC assay was used to measure apoptotic SMMC-7721 cells by flow cytometry. MTT was employed to examine the proliferation of HepG2 cells, which were treated with cisplatin (20 μM) **(E)** or epirubicin (0.55 μg/ml) **(F)** with conditioned medium collected from MSCs which were pretreated with inflammatory cytokines or not. (*Compared with the group that untreated with chemotherapy drugs P < 0.05; #Compared with the group that treated with the conditioned medium collected from MSCs which were pretreated with inflammatory cytokines or not P < 0.05).

**Figure 2:**
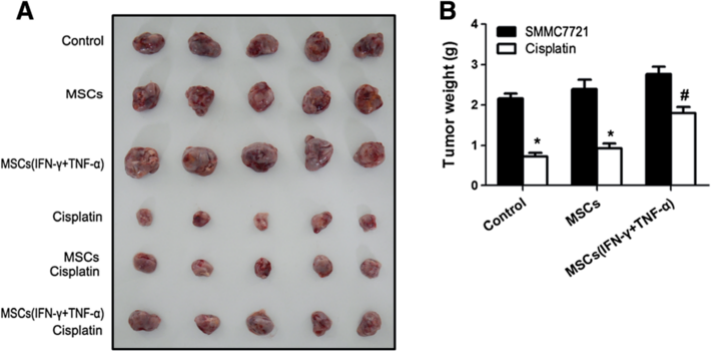
**Inflammatory cytokine-stimulated MSCs enhanced chemoresistance of HCC cells *in vivo. *(A)** MSCs (1 × 10^6^) were pretreated with inflammatory cytokines IFN-γ and TNF-α (20 ng/ml each) for 12 hours and then mixed with SMMC-7721 cells (5 × 10^6^) to perform subcutaneous administration in the nude mice armpit area (five mice for each group). When tumors reached a mean tumor volume of 150 mm^3^, recipients were injected in tumor *in situ* with cisplatin (4 mg/kg) every 3 days. After 27 days of implantation, the animals were sacrificed and tumors were dissected. **(B)** The weight of tumor were measured after been removed from the mice. (*Compared with the group that untreated with chemotherapy drugs P < 0.05; #Compared with the group that MSCs were pretreated with inflammatory cytokines or not P < 0.05).

To study the effect of MSCs on the development of chemoresistance in HCC cells in inflammatory environment, we determined influence of chemotherapeutic agent cisplatin on morphological changes of HCC cell line-SMMC7721 cells in the presence of different types of conditioned media from cultures of MSCs. The results showed that cisplatin effectively inhibited the growth of SMMC7721 cells. This cisplatin-induced inhibition of SMMC7721 cell growth was attenuated when cells were cultured with conditioned medium from the culture of MSCs that were pre-stimulated with a combination of IFN-γ and TNF-α. The development of chemoresistance was not observed in SMMC7721 cells when they were cultured with conditioned medium from the culture of untreated MSCs or from the culture of MSCs pretreated with either IFN-γ or TNF-α alone (Figure 1A). SMMC7721 cell proliferation was also determined by MTT assay to verify the development of chemoresistance in these cells. The results confirmed that conditioned medium from the culture of MSCs pre-stimulated with the combination of IFN-γ and TNF-α could effectively improve SMMC7721 cell proliferation in the presence of the chemotherapeutic agent. In contrast, SMMC7721 cell proliferation was significantly inhibited by cisplatin or epirubicin in control groups (Figure 1B and C). Cisplatin treatment effectively enhanced apoptosis in SMMC7721 cells (Figure 1D). Conditioned medium of MSCs pre-stimulated with the combination of IFN-γ and TNF-α caused a significant reduction of cisplatin-induced apoptosis in cultured SMMC7721 cells. We also employed the culture model of HepG2 cells to study chemoresistance induced by MSCs. The results demonstrated that conditioned medium of MSCs pre-stimulated with the combination of IFN-γ and TNF-α could effectively protect HepG2 cells from cell toxicity caused by cisplatin or epirubicin (Figure 1E-F). We further examined the MSCs-induced chemoresistance in HCC cells in the nude mouse model. The results showed that MSCs pretreated with the combination of IFN-γ and TNF-α could lead to resistance to chemotherapy in HCC cells *in vivo* (Figure 2A and B).

### Inflammatory cytokine-stimulated MSCs induced autophagy in hepatocellular carcinoma cells

**Figure 3:**
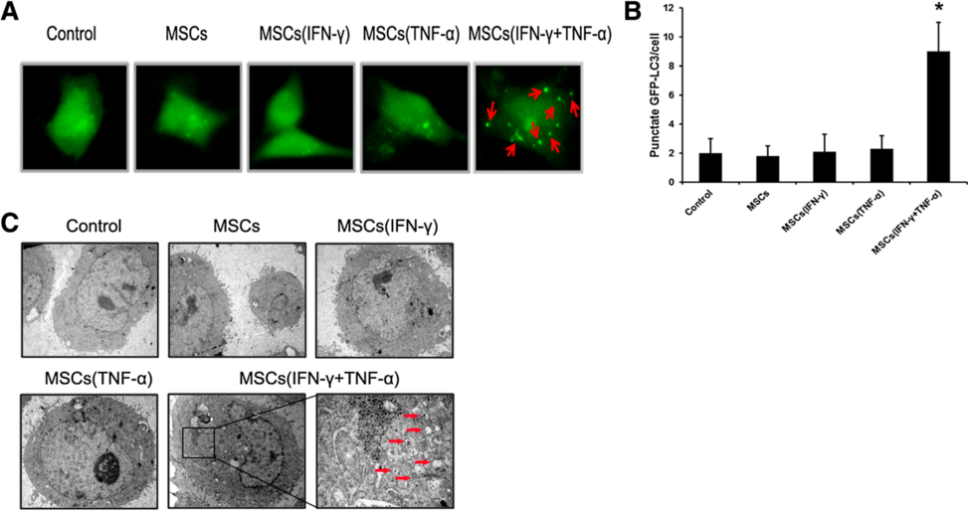
**Conditioned medium from the culture of inflammatory cytokine-stimulated MSCs induced autophagy in HCC cells. (A)** SMMC-7721 cells were transfected with GFP-tagged LC3; after 24 hours transfection, cells were incubated with contidioned medium collected from MSCs which were pretreated with inflammatory cytokines or not. Images were taken under a fluorescence microscope. Arrows show the punctate GFP-LC3 in the cytoplasm. **(B)** The number of punctate GFP-LC3 in each cell of SMMC-7721 was counted and at least 100 cells were included for each group. The results were shown as means (±SD) (*p < 0.05). **(C)** Electron micrographs showing the ultrastructure of SMMC-7721 cells with or without conditioned medium collected from MSCs pretreated with inflammatory cytokines or not. Arrows indicate the autophagic vacuoles in the cytoplasm. Magnification, ×10,000.

In previous studies, we have demonstrated that autophagy contributes to the resistance of HCC cells to chemotherapeutic agents [21]. We hypothesized that MSCs might induce autophagy of HCC cells in inflammatory environment. Analysis of autophagy in SMMC7721 cells was performed using an expression vector encoding GFP-LC3 which is concentrated in autophagic vacuoles, resulting in punctate fluorescence within cells undergoing autophagy. As shown in Figure 3A and B, MSCs pretreated with the combination of IFN-γ and TNF-α effectively induced autophagy in SMMC7721 cells, which exhibited a significantly high number of punctate GFP. In contrast, SMMC7721 cells in control groups showed primarily diffused fluorescence. In order to confirm the above observation, we employed transmission electron microscopy to detect the autophagy in SMMC7721 cells. The results demonstrated that the occurrence of autophagy could be observed in SMMC7721 cells when cultured with conditioned medium from the culture of MSCs pre-stimulated with the combination of IFN-γ and TNF-α (Figure 3C).

### Inhibition of autophagy restored the sensitivity of HCC cells to chemotherapy

**Figure 4:**
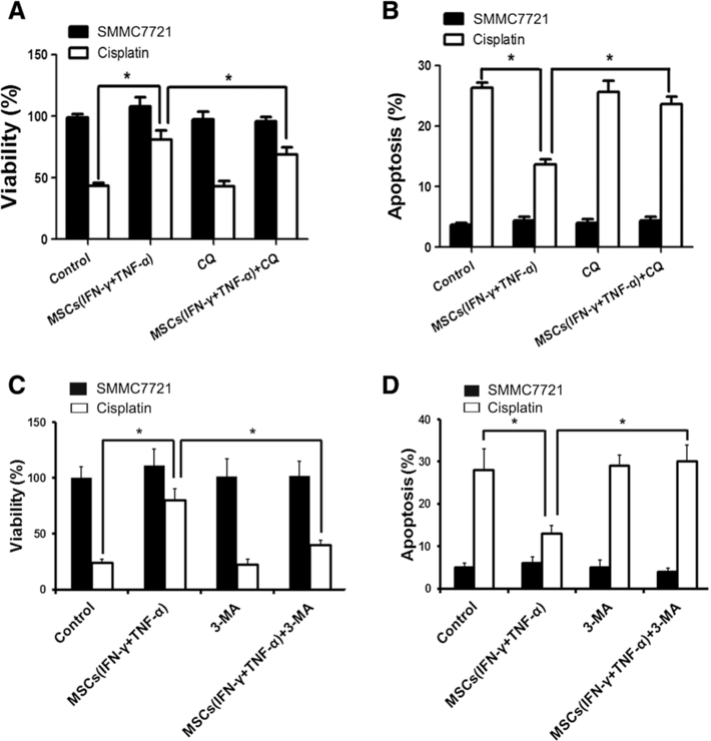
**Inhibition of autophagy restored HCC cell sensitivity to chemotherapy. (A)** SMMC-7721 cells (1 × 10^4^/well) were cultured in a 96-well plate with an existence of cisplatin (20 μM) and the conditioned medium collected from MSCs which were pretreated with inflammatory cytokines or not were added in SMMC-7721 culture medium for 24 hours. The occurrence of autophagy was inhibited by autophagy inhibitor-CQ (10 μM). MTT was employed to examine the proliferation of SMMC-7721 cells. **(B)** SMMC-7721 cells were cultured in a 6-well plate with an existence of cisplatin (20 μM) and the conditioned medium collected from MSCs which were pretreated with inflammatory cytokines or not were added in SMMC-7721 culture medium for 24 hours. The occurrence of autophagy was inhibited by autophagy inhibitor-CQ (10 μM). PI/Annexin V-FITC assay was used to measure apoptotic SMMC-7721 cells ells by flow cytometry. **(C)** SMMC-7721 cells (1 × 10^4^/well) were cultured in a 96-well plate with an existence of cisplatin (20 μM) and the conditioned medium collected from MSCs which were pretreated with inflammatory cytokines or not were added in SMMC-7721 culture medium for 24 hours. The occurrence of autophagy was inhibited by autophagy inhibitor-3-MA (2 mM). MTT was employed to examine the proliferation of SMMC-7721 cells. **(D)** SMMC-7721 cells were cultured in a 6-well plate with an existence of cisplatin (20 μM) and the conditioned medium collected from MSCs which were pretreated with inflammatory cytokines or not were added in SMMC-7721 culture medium for 24 hours. The occurrence of autophagy was inhibited by autophagy inhibitor-3-MA (2 mM). PI/Annexin V-FITC assay was used to measure apoptotic SMMC-7721 cells ells by flow cytometry. (*P < 0.05).

In order to further verify that occurrence of autophagy in HCC cells lead to enhancement of chemoresistance in HCC cells, we examined the recurrence of HCC cell sensitivity to chemotherapy in the presence of autophagy inhibitors. The results showed that the autophagy inhibitor CQ and 3-MA could effectively restore the sensitivity of HCC cells to chemotherapeutic agents. Addition of autophagy inhibitors to the cell culture resulted in inhibition of proliferation and increase in apoptosis in HCC cells following treatment with chemotherapeutic agents (Figure 4A-D).

### Inflammatory cytokines induced overexpression of TGF-β by MSCs

**Figure 5:**
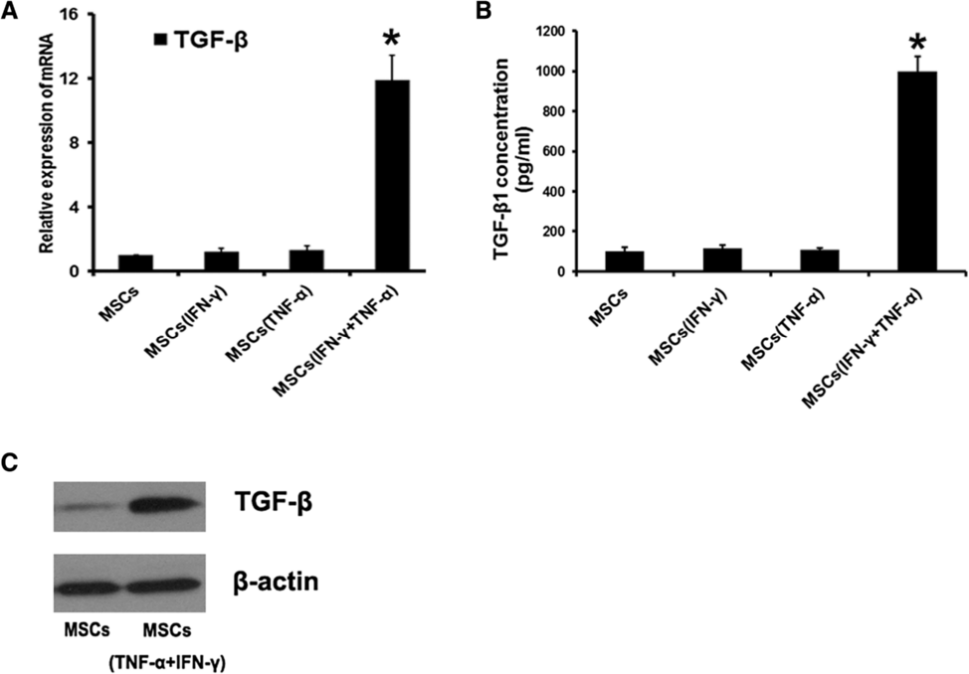
**Inflammatory cytokines induced overexpression of TGF-β by MSCs.** MSCs were pretreated with inflammatory cytokines IFN-γ and TNF-α (20 ng/ml each) for 12 hours. **(A)** TGF-β mRNA in MSCs was assayed by real-time PCR. **(B)** The amount of TGF-β1 in conditioned medium was detected by ELISA. **(C)** The expression of TGF-β in protein level was examined by western blot. (*P < 0.05).

Several studies have suggested that TGF-β plays an important role in induction of autophagy [22-24]. Therefore, we examined TGF-β expression by MSCs in response to IFN-γ and TNF-α stimulation. As shown in Figure 5, stimulation with the combination of IFN-γ and TNF-α caused a significant up-regulation of TGF-β expression at both mRNA and protein levels in MSCs.

### Inhibition of TGF-β expression by MSCs diminished the ability of MSCs to induce autophagy and chemoresistance in HCC cells

**Figure 6:**
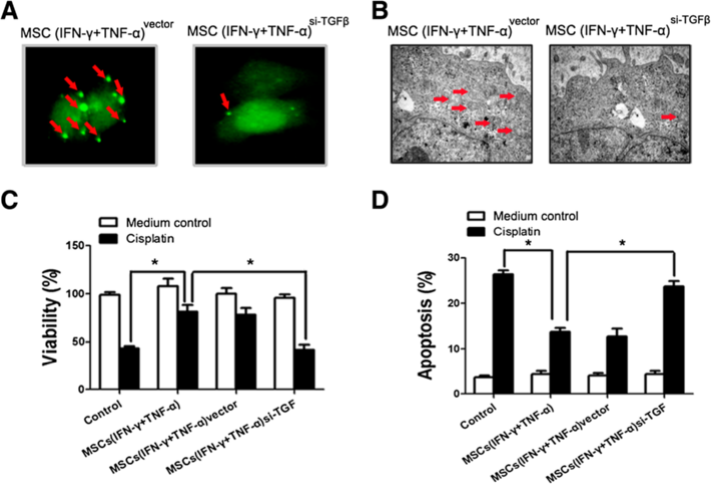
**Inhibition of TGF-β expression by MSCs diminished the ability of MSCs in inducing autophagy and chemoresistance in HCC cells. (A)** MSCs were transfected with si-TGFβ and then been stimulated by both IFN-γ and TNF-α for 12 hours. The conditioned medium was collected for further investigation. SMMC-7721 cells were transfected with GFP-tagged LC3; after 24 hours transfection, cells were incubated with contidioned medium collected from MSCs^si-TGFβ^. Images were taken under a fluorescence microscope. Arrows show the punctate GFP-LC3 in the cytoplasm. **(B)** Electron micrographs showing the ultrastructure of SMMC-7721 cells with or without conditioned medium collected from MSCs pretreated with inflammatory cytokines or not. Arrows indicate the autophagic vacuoles in the cytoplasm. Magnification, ×10000. **(C)** SMMC-7721 cells (1 × 10^4^/well) were cultured in a 96-well plate with an existence of cisplatin (20 μM) and the conditioned medium collected from MSCs^si-TGFβ^ was added in SMMC-7721 culture medium for 24 hours. MTT was employed to examine the proliferation of SMMC-7721 cells. **(D)** SMMC-7721 cells were cultured in a 6-well plate with an existence of cisplatin (10 μg/mL) and the conditioned medium collected from MSCs^si-TGFβ^ was added in SMMC-7721 culture medium for 24 hours. PI/Annexin V-FITC assay was used to measure apoptotic SMMC-7721 cells by flow cytometry. (*P < 0.05).

In order to define the role of TGF-β in MSC-mediated occurrence of autophagy and development of chemoresistance in HCC cells, TGF-β expression by MSCs in response to IFN-γ plus TNF-α stimulation was knockdown by transfecting MSCs with siRNA against TGF-β mRNA. Conditioned medium from the culture of TGF-β knockdown MSCs failed to induce formation of autophagy in HCC cells (Figure 6A and B). Furthermore, blockade of TGF-β expression by MSCs diminished the ability of these MSCs to induce chemoresistance in HCC cells (Figure 6C and D). These results indicate that up-regulation of TGF-β expression by MSCs in inflammatory microenvironment plays a key role in inducing autophagy and development of chemoresistance in HCC cells.

## Discussion

Autophagy is a survival mechanism for both prokaryotic and eukaryotic cells in the nutrient deficient environment [25-27]. Energy, amino acids, and other precursor molecules for maintaining cellular homeostasis and facilitating cell survival are produced through this process [28-30]. It has been reported that autophagy associates with various physiological and pathological processes, including differentiation, tumorigenesis, chemoresistance and adaptation to changed environmental conditions [31]. The process of autophagy includes three steps: (1) autophagosome formation, (2) lysosomal fusion with the autophagosome, and (3) lysosomal degradation to produce precursor molecules, such as amino acids and fatty acids, to be reutilized for de novo synthesis of macromolecules and generation of energy [32]. Autophagy is activated by hypoxia, starvation and TGF-β1 [21,33,34]. On the other hand, the occurrence of autophagy can be inhibited not only by the key factor in autophagy associated signal pathway such as Atg 7 and Beclin 1 but also by autophagy inhibitor CQ and 3-MA. 3-MA serves as an inhibitor of phosphatidylinositol 3-kinase, which blocks autophagosome formation to inhibit autophagy [35]. CQ is a lysosomotropic drug that impairs autophagic protein degradation. CQ blocks the last step of the autophagy pathway, which leads to the accumulation of autophagosomes [36,37].

MSCs play an important role in the pathogenesis of various degenerative diseases and immune disorders. This cell population is a potential target for correcting aberrant immune reactions. Studies have shown that MSCs can be induced and expanded ex vivo. These precursor cells can terminally differentiate into osteoblasts, chondrocytes, adipocytes, myotubes, neural cells and hematopoietic supporting stroma [9,10,38]. In previous studies, we have demonstrated that in tumor inflammatory microenvironment, MSCs may exert immunosuppressive function through iNOS to protect tumor cell from immune surveillance [39]. Furthermore, MSCs have been shown to promote tumor growth by enhancing tumor angiogenesis via HIF-1α-VEGF signalling pathway in the colon cancer model [40]. In this study, we observed that in inflammatory microenvironment, MSCs could effectively induce chemoresistance in HCC cell line cells.

In previous studies, we have demonstrated that autophagy decreases the sensitivity of hepatoma cells to chemotherapeutic agents by affecting their apoptotic potential [21]. In addition, we have shown that autophagy activated by hypoxia mediates the tolerance of hepatocellular carcinoma cells to nutrient deprivation, which is dependent on the activity of Beclin 1 [41]. In this study, we found that MSC-induced chemoresistance in HCC cell line cells was associated with the occurrence of autophagy, a protective mechanism of HCC cells to resist chemotherapeutic agents. Stimulation with the combination of IFN-γ and TNF-α could provoke TGF-β expression by MSCs. The enhancement of chemoresistance in HCC cell line cells was in a TGF-β dependent manner. Our results suggest that overexpression of TGF-β by MSCs in the HCC inflammatory microenviroment may favor the development of chemoresistance in HCC cells by inducing autophagy.

TGF-β plays an important role in regulating cell growth, differentiation and migration. TGF-β has been reported to induce autophagy in normal bovine mammary epithelial BME-UV1 cells [12]. Furthermore, TGF-β also induced autophagy not only in some mammary carcinoma cells but also in human hepatocellular carcinoma cells. Accumulation of autophagosomes and conversion of LC3 from type1 to type2 induced by TGF-β results in degradation of long-lived proteins. TGF-β upregulates the expression of ATG5, ATG7, BECLIN1, and death-associated protein kinase (DAPK) in mRNA level [6]. Recently, Roodhart and colleagues have reported that endogenous MSCs become activated during treatment with platinum analogs and secrete factors which protect tumor cells against a range of chemotherapeutic agents [42]. Our results reveal another important fact that tumor inflammatory microenvironment is a key player in activating MSCs to induce chemoresistance of HCC cells. Inflammation is a fundamental feature during the development of hepatocellular carcinoma, which exists not only within the tumor tissue but also in tissues surrounding the tumor. MSCs in inflammatory microenvironment may persistently promote the development of chemoresistance in HCC cells during tumor growth. One mechanism underlying MSC-promoted development of chemoresistance in HCC cells is via their overexpression of TGF-β in response to inflammatory stimuli in the tumor microenvironment. Inhibition of MSC activation in the inflammatory microenvironment may serves as a potential approach to improve chemosensitivity of HCC cells.

## Conclusions

Taken together, these results suggest that increase in TGF-β expression by MSCs in the inflammatory microenvironment of HCC promotes the development of chemoresistance in HCC cells.

## Abbreviations

MSCs: Mesenchymal stem cells; HCC: Hepatocellular carcinoma; TGF-β: Transforming growth factor-β.

## Competing interests

The authors declare that they have no conflicts of interests.

## Authors’ contributions

ZPH: Conception and design, data analysis and interpretation, manuscript writing. YYJ: Conception and design, collection and/or assembly of data, manuscript writing. YX: Collection and/or assembly of data, data analysis and interpretation. SSZ: Conception and design, data analysis and interpretation. JH: Administrative support, collection and/or assembly of data. YM: Provision of study material or patients, collection and/or assembly of data, data analysis and interpretation. FHY: Provision of study material or patients, collection and/or assembly of data. XQL: Collection and/or assembly of data, data analysis and interpretation. MCW: Data analysis and interpretation. PZ: Conception and design, data analysis and interpretation. LXW: Conception and design, financial support, data analysis and interpretation, final approval of manuscript. All authors read and approved the final manuscript.
